# GENDER DIFFERENCES IN RURAL-URBAN MIGRATION AND ITS IMPACT ON MENTAL HEALTH IN LATER LIFE

**DOI:** 10.1093/geroni/igac059.3042

**Published:** 2022-12-20

**Authors:** Jingwen Zhang, James Nazroo, Nan Zhang

**Affiliations:** The University of Manchester, Manchester, England, United Kingdom; The University of Manchester, Manchester, England, United Kingdom; The University of Manchester, Manchester, England, United Kingdom

## Abstract

Although rural-to-urban migration has been well researched, how gender shapes processes and outcomes, including later life health outcomes, has not been thoroughly investigated. Guided by a life course perspective, this study explores gender differences in rural-urban migration patterns and its association with mental health in later life among Chinese older adults. Exploiting rich life history data from the China Health and Retirement Longitudinal Study, we employ sequence analysis to identify the typical migration trajectories of Chinese older adults. Moderated mediation analysis is then used to examine gender-specific health pathways linking migration trajectories and later-life mental health. The results indicate that: men and women follow different migration trajectories across the life course. Men are more likely to migrate between rural and urban areas, and to migrate multiple times. Rural migrants who settled in urban regions have better mental health in later life than return migrants or rural non-migrants; the gender gap in mental health is marginally smaller among early urban settlers than rural non-migrants. Household income and Hukou conversion mediate the relationship between migration trajectories and later-life mental health among older people of rural origin. Household income in later life has stronger mediation effects for migrant men than for migrant women. The study suggests that a life course perspective and awareness of gender dynamics should be incorporated in policymaking to reduce the rural-urban divide and gender-based inequality in mental health.

